# Supplementary material for: Effects of exercise training with blood flow restriction on vascular function in adults: a systematic review and meta-analysis
Source: PeerJ. 2021 Jul 7;9:e11554. doi: 10.7717/peerj.11554 (PMC8272459; doi:10.7717/peerj.11554)
Supplement: Supplemental Information 2 [file peerj-09-11554-s002.docx]

| Database (website) | Search terms | Number of manuscripts |
| --- | --- | --- |
| PubMed (<https://pubmed.ncbi.nlm.nih.gov/>) | ("blood flow restriction" OR "blood flow occlusion" OR "vascular occlusion" OR "kaatsu training") AND ("vascular function" OR "endothelial function” OR “arterial stiffness” OR “pulse wave velocity” OR “flow-mediated dilatation” OR “VEGF1” OR “nitric oxide”) | 183 |
