# Supplementary material for: Effects of exercise training with blood flow restriction on vascular function in adults: a systematic review and meta-analysis
Source: PeerJ. 2021 Jul 7;9:e11554. doi: 10.7717/peerj.11554 (PMC8272459; doi:10.7717/peerj.11554)
Supplement: Supplemental Information 3 — RCT, Randomized controlled trial; CT, Controlled trial; RCOT, Randomized cross-over trial; SD, Standard deviation; m, Male; f, Female; HG, Handgrip; UL, Upper limbs; LL, Lower limbs; BFR, Blood flow restriction; HI, High intensity; MI, Moderate intensity; LI, Low-intensity. [file peerj-09-11554-s003.docx]

| First author Year | Study design | | | | Comparator (no BFR) | | | Population group | Age mean (SD) | n (m/f) | Exercise modality | | | |
| --- | --- | --- | --- | --- | --- | --- | --- | --- | --- | --- | --- | --- | --- | --- |
|  | RCT | CT | RCOT | Other | HI | MI | LI |  |  |  | Treadmill walking | Dynamic resistance training | HG | Other |
| Ramis 2020 | √ |  |  |  | √ |  |  | Healthy adults | 23.9 (2.7) | 28 (28/0) |  | √ |  |  |
| Credeur 2019 |  |  | √ |  |  | √ |  | Healthy adults | 25 (2) | 15 (15/0) |  |  | √ |  |
| Kambic 2019 | √ |  |  |  |  |  | √ | Coronary artery disease | 60.5 (2.4) | 24 (18/6) |  | √ |  |  |
| Lopes 2019 |  |  |  | √ |  |  | √ | Sarcopenia | 91 | 1 (1/0) |  | √ |  |  |
| Mouser 2019 | √ |  |  |  | √ |  | √ | Healthy adults | 21.5 (2.4) | 40 (20/20) |  | √ |  |  |
| Barili 2018 |  |  | √ |  | √ |  | √ | Hypertensive older women | 67.2 (3.7) | 16 (0/16) | √ |  |  |  |
| Boeno 2018 |  |  | √ |  | √ |  | √ | Healthy adults | 23.7 (3.4) | 11 (11/0) |  | √ |  |  |
| Karabulut 2018 |  |  | √ |  |  |  | √ | Healthy adults | 22.6 (2.2) | 8 (8/0) |  |  |  | √ |
| Natsume 2018 |  |  | √ |  |  |  | √ | Healthy adults | 26 (1) | 8 (8/0) |  |  |  | √ |
| Sardeli 2017 |  |  | √ |  | √ |  | √ | Healthy older adults | 64.3 (5.1) | 21 (9/12) |  | √ |  |  |
| Paiva 2016 |  |  | √ |  |  | √ |  | Healthy adults | 28 (5.8) | 9 (9/0) |  |  | √ |  |
| Shimizu 2016 | √ |  |  |  |  |  | √ | Healthy older adults | 71 (4) | 40 (33/7) |  | √ |  |  |
| Yasuda 2016 | √ |  |  |  |  | √ |  | Healthy older women | 70 (6) | 30 (0/30) |  | √ |  |  |
| Yasuda 2015a | √ |  |  |  |  |  | √ | Healthy older women | 69.5 (6.5) | 14 (0/14) |  | √ |  |  |
| Yasuda 2015b |  | √ |  |  |  |  | √ | Healthy older adults | 69.9 (5.6) | 17 (3/14) |  | √ |  |  |
| Fahs 2014 | √ |  |  |  |  |  | √ | Healthy adults | 55 (7) | 16 (11/5) |  | √ |  |  |
| Ozaki 2013 | √ |  |  |  | √ |  |  | Healthy adults | 23 (0) | 19 (19/0) |  | √ |  |  |
| Fahs 2012 |  | √ |  |  | √ | √ |  | Healthy adults | 21 (0) | 46 (46/0) |  | √ |  |  |
| Hunt 2012 | √ |  |  |  |  | √ |  | Healthy adults | 26 (4) | 9 (9/0) |  |  | √ |  |
| Larkin 2012 |  |  | √ |  |  |  | √ | Healthy adults | 22 (1) | 6 (6/0) |  | √ |  |  |
| Clark 2011 | √ |  |  |  | √ |  |  | Healthy adults | 24.3 (1.7) | 16 (14/2) |  | √ |  |  |
| Figueroa 2011 |  |  | √ |  |  |  | √ | Healthy adults | 22 (2) | 23 (11/12) |  | √ |  |  |
| Patterson 2011 | √ |  |  |  |  |  | √ | Healthy older adults | 67 (3) | 10 (8/2) |  | √ |  |  |
| Credeur 2010 | √ |  |  |  |  | √ |  | Healthy adults | 22 (1) | 12 (5/7) |  |  | √ |  |
| Patterson 2010 | √ |  |  |  |  | √ | √ | Healthy adults | 22 (3) | 16 (0/16) |  | √ |  |  |
| Renzi 2010 |  |  | √ |  |  |  | √ | Healthy adults | 26 (1) | 17 (11/6) | √ |  |  |  |

RCT, Randomized controlled trial; CT, Controlled trial; RCOT, Randomized cross-over trial; SD, Standard deviation; m, Male; f, Female; HG, Handgrip; UL, Upper limbs; LL, Lower limbs; BFR, Blood flow restriction; HI, High intensity; MI, Moderate intensity; LI, Low-intensity

**TABLE CONTINUING**

| First author Year | Exercised limbs | | Length of intervention (weeks) | | | | | |
| --- | --- | --- | --- | --- | --- | --- | --- | --- |
|  | UL | LL | Single session | 4 | 6 | 8 | 12 | 16 |
| Ramis 2020 | √ | √ |  |  |  | √ |  |  |
| Credeur 2019 | √ |  | √ |  |  |  |  |  |
| Kambic 2019 |  | √ |  |  |  | √ |  |  |
| Lopes 2019 | √ | √ |  |  |  |  | √ |  |
| Mouser 2019 | √ | √ |  |  |  |  |  | √ |
| Barili 2018 |  | √ | √ |  |  |  |  |  |
| Boeno 2018 | √ | √ | √ |  |  |  |  |  |
| Karabulut 2018 | √ | √ | √ |  |  |  |  |  |
| Natsume 2018 |  | √ | √ |  |  |  |  |  |
| Sardeli 2017 |  | √ | √ |  |  |  |  |  |
| Paiva 2016 | √ |  | √ |  |  |  |  |  |
| Shimizu 2016 | √ | √ |  | √ |  |  |  |  |
| Yasuda 2016 |  | √ |  |  |  |  | √ |  |
| Yasuda 2015a | √ |  |  |  |  |  | √ |  |
| Yasuda 2015b | √ |  |  |  |  |  | √ |  |
| Fahs 2014 |  | √ |  |  | √ |  |  |  |
| Ozaki 2013 | √ |  | √ |  | √ |  |  |  |
| Fahs 2012 | √ | √ |  |  | √ |  |  |  |
| Hunt 2012 | √ |  |  | √ |  |  |  |  |
| Larkin 2012 |  | √ | √ |  |  |  |  |  |
| Clark 2011 |  | √ |  | √ |  |  |  |  |
| Figueroa 2011 |  | √ | √ |  |  |  |  |  |
| Patterson 2011 |  | √ |  | √ |  |  |  |  |
| Credeur 2010 | √ |  |  | √ |  |  |  |  |
| Patterson 2010 |  | √ |  | √ |  |  |  |  |
| Renzi 2010 |  | √ | √ |  |  |  |  |  |

RCT, Randomized controlled trial; CT, Controlled trial; RCOT, Randomized cross-over trial; SD, Standard deviation; m, Male; f, Female; HG, Handgrip; UL, Upper limbs; LL, Lower limbs; BFR, Blood flow restriction; HI, High intensity; MI, Moderate intensity; LI, Low-intensity
