# Supplementary material for: Effects of exercise training with blood flow restriction on vascular function in adults: a systematic review and meta-analysis
Source: PeerJ. 2021 Jul 7;9:e11554. doi: 10.7717/peerj.11554 (PMC8272459; doi:10.7717/peerj.11554)
Supplement: Supplemental Information 5 [file peerj-09-11554-s005.docx]

| First author Year | Assessment to prescribe exercise intensity | | | | Exercise intensity | Number of sets | | | | Total number of repetitions | Exercise duration (minutes) | Cuff width (cm)* | Approach to determine restriction pressure | | | |
| --- | --- | --- | --- | --- | --- | --- | --- | --- | --- | --- | --- | --- | --- | --- | --- | --- |
|  |  |  |  |  |  |  |  |  |  |  |  |  | Individualized | | | Non-individualized |
|  | 1RM | VO_2max_ | MVC | Other |  | ≤2 | 3 | 4 | ≥5 |  |  |  | AOP | SBP | LC | Pre-determined (mmHg) |
| Ramis 2020 | √ |  |  |  | 30% |  |  | √ |  | UL: 21  LL: 23 | NR | UL: 14  LL: 16 |  | UL: 20 mmHg lower than SBP; LL: 40 mmHg higher than UL |  |  |
| Credeur 2019 |  |  | √ |  | 40% | √ |  |  |  | – | 5 | 10 |  |  |  | 80 – 100 |
| Kambic 2019 | √ |  |  |  | 30 – 40% |  | √ |  |  | 30 | NR | 23 |  | 15-20 mmHg higher than SBP |  |  |
| Lopes et 2019 | √ |  |  |  | 30% |  | √ |  |  | 30 | 12 | 11 |  | 50% SBP |  |  |
| Mouser 2019 | √ |  |  |  | 15% |  |  | √ |  | Until failure | NR | NR | 40% |  |  |  |
| Barili 2018 |  | √ |  |  | 30% |  |  |  |  | – | NR | 9.5 |  | 130% of SBP |  |  |
| Boeno 2018 | √ |  |  |  | 30% |  |  | √ |  | Until failure | NR | NR |  | UL: 20 mmHg higher than SBP LL: 20 mmHg lower than SBP |  |  |
| Karabulut 2018 |  |  |  | √ | – |  |  |  | √ | – | NR | NR |  | SBP x 1.44 |  |  |
| Natsume 2018 |  |  | √ |  | 10% |  |  | √ |  | – | 20 | 10.5 |  |  | <50 cm: 140 mmHg  ≥55 cm: 160 mmHg |  |
| Sardeli 2017 | √ |  |  |  | 30% |  |  | √ |  | 75 | NR | 17.5 | 50% |  |  |  |
| Paiva 2016 |  |  | √ |  | 60% |  |  |  |  | NR | 20 | NR |  |  |  | 80 |
| Shimizu 2016 | √ |  |  |  | 20% |  | √ |  |  | 60 | NR | UL: 7  LL: 10 |  | UL: Brachial SBP  LL: Femoral SBP |  |  |
| Yasuda 2016 |  |  |  | √ | – |  |  | √ |  | 75 | 11 | 5 |  |  |  | 120 – 200 |
| Yasuda 2015a |  |  |  | √ | – |  |  | √ |  | 75 | 9.5 | 3 |  |  |  | 120 – 200 |
| Yasuda 2015b |  |  |  | √ | – |  |  | √ |  | 75 | 11 | 3 |  |  |  | 120 – 200 |
| Fahs 2014 | √ |  |  |  | 30% | √ | √ | √ |  | Until failure | NR | 5 | 50 – 80% AOP OR 150 – 240, whichever was higher |  |  | 50 – 80% AOP OR 150 – 240, whichever was higher |
| Ozaki 2013 | √ |  |  |  | 30% |  |  | √ |  | 75 | NR | 3 |  |  |  | 160 |
| Fahs 2012 | √ |  |  |  | 20% |  |  | √ |  | 75 | 12 | 10.5 |  |  |  | 160 - 200 |
| Hunt 2012 | √ |  |  |  | 40% |  | √ |  |  | Until failure | 8.5±2.7 | 13 |  |  |  | 80 |
| Larkin 2012 | √ |  |  |  | 40% |  |  |  | √ | 120 | NR | NR |  |  |  | 220 |
| Clark 2011 | √ |  |  |  | 30% |  | √ |  |  | 24 – 36 | NR | 6 |  | 130% of SBP |  |  |
| Figueroa 2011 | √ |  |  |  | 40% |  | √ |  |  | Until failure | NR | NR |  |  |  | 100 |
| Patterson 2011 | √ |  |  |  | 25% |  | √ |  |  | Until failure | NR | 5-8 |  |  |  | 110 |
| Credeur 2010 |  |  | √ |  | 60% | √ |  |  |  | – | 20 | NR |  |  |  | 80 |
| Patterson 2010 | √ |  |  |  | 25% |  | √ |  |  | Until failure | NR | NR |  |  |  | 110 |
| Renzi 2010 |  |  |  | √ | 2 miles/h |  |  |  | √ | – | 13 | NR |  |  |  | 160 |

1RM, One-repetition maximum; VO_2max_, Maximum Oxygen consumption; MVC, Maximum voluntary contraction; –, Not applicable; NR, Not reported; UL, Upper limbs; LL, Lower limbs; *, All studies used a cuff to promote blood flow restriction; AOP, Arterial occlusion pressure; SBP, Systolic blood pressure; LC, Limb circumference.
