# Supplementary material for: Effects of exercise training with blood flow restriction on vascular function in adults: a systematic review and meta-analysis
Source: PeerJ. 2021 Jul 7;9:e11554. doi: 10.7717/peerj.11554 (PMC8272459; doi:10.7717/peerj.11554)
Supplement: Supplemental Information 6 — BFRE, Blood flow restricted exercise; K, Number of studies in that category; B, Regression estimate; 95% CI, 95 Percent Confidence Limits; CT, Controlled trial; RCT, Randomized controlled trial; RCOT, Randomized cross-over trial; *, Statistically significant difference (p≤0.05); Cuff width not included in meta-regression for endothelial function (less than 10 studies); Mean pressure, The mean value reported for the pressure used during exercise; Cuff width, Mean pressure and Study quality are continuous variables. [file peerj-09-11554-s006.docx]

| Predictor variable |  | Vascular structure | | | | Endothelial function | | | |
| --- | --- | --- | --- | --- | --- | --- | --- | --- | --- |
|  |  | K | B | 95% CI | p | K | B | 95% CI | p |
| Study design | CT | 2 |  |  |  | 2 |  |  |  |
|  | RCT | 8 | 0.04 | (-1.22, 1.30) | 0.94 | 9 | -0.86 | (-2.84, 1.10) | 0.39 |
|  | RCOT | 5 | -0.24 | (-1.55, 1.06) | 0.71 | 2 | -2.23 | (-5.40, -0.42) | 0.02 |
| Study quality (TESTEX tool) |  | 15 | 0.01 | (-2.25, 2.52) | 0.95 | 13 | -0.06 | (-0.50, 0.39) | 0.8 |
| Population group | Healthy adults | 11 |  |  |  | 8 |  |  |  |
|  | Healthy older adults | 4 | -0.13 | (-1.02, 0.74) | 0.75 | 4 | -0.94 | (-3.93, 2.05) | 0.54 |
|  | Clinical population | 0 | - | - | - | 1 | 1.6 | (-1.08, 4.27) | 0.24 |
| Exercise modality | Handgrip | 3 |  |  |  | 4 |  |  |  |
|  | Treadmill walking | 1 | 1.33 | (-0.18, 2.86) | 0.08 | 1 | -2.62 | (-4.18, -1.06) | 0.001 |
|  | Dynamic resistance training | 10 | 0.69 | (-0.25, 1.62) | 0.15 | 8 | 1.64 | (0.83, 2.45) | <.0001 |
|  | Others | 1 | -0.03 | (-1.19, 0.47) | 0.39 | 0 | - | - | - |
| Exercised limbs | Upper and lower limbs | 3 |  |  |  | 2 |  |  |  |
|  | Upper limbs | 7 | -0.04 | (-1.13, 1.04) | 0.94 | 6 | -2.22 | (-4.23, -0.19) | 0.03 |
|  | Lower limbs | 5 | 0.21 | (-0.93, 1.34) | 1.35 | 5 | -1.36 | (-3.44, 0.71) | 0.2 |
| Length of intervention | Single session | 6 |  |  |  | 3 |  |  |  |
|  | 4 weeks | 2 | 0.54 | (-1.04, 2.13) | 0.5 | 4 | 0.48 | (-1.47, 2.43) | 0.63 |
|  | 6 weeks | 3 | 0.5 | (-0.86, 1.86) | 0.47 | 1 | 2.64 | (-0.30, 5.58) | 0.08 |
|  | 8 weeks | 0 | - | - | - | 1 | 1.38 | (-0.92, 3.69) | 0.24 |
|  | 12 weeks | 3 | -0.05 | (-1.00, 0.90) | 0.92 | 2 | 0.03 | (-1.62. 1.67) | 0.97 |
|  | 16 weeks | 1 | 0.1 | (-1.79, 1.99) | 0.92 | 0 | - | - | - |
| Length of intervention (Acute *versus* longer studies grouped) | Single session | 6 |  |  |  | 3 |  |  |  |
|  | ≥4 weeks | 9 | 0.07 | (-0.72, 0.86) | 0.86 | 10 | 1.92 | (0.39, 3.46) | 0.01 |
| Number of sets per exercise | ≤2 | 2 |  |  |  | 2 |  |  |  |
|  | 3 | 8 | -0.46 | (-1.42, 0.50) | 0.35 | 5 | 1.66 | (0.21, 3.11) | 0.02 |
|  | 4 | 2 | -0.29 | (-1.51, 0.93) | 0.65 | 4 | 1.54 | (0.03, 3.05) | 0.05 |
|  | ≥5 | 0 | - | - |  | 1 | -2.5 | (-4.69, -0.30) | 0.03 |
| Cuff width |  | 10 | 0 | (-0.12, 0.11) | 0.95 | - | - | - | - |
| Approach to determine restriction pressure | Pre-determined | 10 |  |  |  | 3 |  |  |  |
|  | Arterial occlusion pressure | 3 | 0.01 | (-0.88, 0.89) | 0.98 | 0 | - | - | - |
|  | Systolic blood pressure | 2 | 0.45 | (-1.02, 1.92) | 0.54 | 10 | 1.33 | (-0.24, 2.90) | 0.1 |
|  | Limb circumference | 0 | - | - | - | 0 | - | - | - |
| Mean pressure |  | 10 | 0.01 | (-0.01. 0.02) | 0.38 | 11 | 0 | (-2.84, 2.25) | 0.72 |

BFRE, Blood flow restricted exercise; K, Number of studies in that category; B, Regression estimate; 95% CI, 95 Percent Confidence Limits; CT, Controlled trial; RCT, Randomized controlled trial; RCOT, Randomized cross-over trial; *, Statistically significant difference (p≤0.05); Cuff width not included in meta-regression for endothelial function (less than 10 studies); Mean pressure, The mean value reported for the pressure used during exercise; Cuff width, Mean pressure and Study quality are continuous variables
