# Supplementary material for: Effects of exercise training with blood flow restriction on vascular function in adults: a systematic review and meta-analysis
Source: PeerJ. 2021 Jul 7;9:e11554. doi: 10.7717/peerj.11554 (PMC8272459; doi:10.7717/peerj.11554)
Supplement: Supplemental Information 7 — –, Not applicable NR, Not reported; *, Percentage of exercise sessions attended by the subjects; **, Percentage of subjects who gave consent and were randomized, but did not have their data included in the analysis; ***, Reported adverse event was sensation of leg numbness in the first moments of exercise with BFR, which did not persist beyond the first minute of exercise. [file peerj-09-11554-s007.docx]

| First author Year | Adverse events | | | Number (%) of people who had adverse events | Adherence to sessions* | Dropout rate** |
| --- | --- | --- | --- | --- | --- | --- |
|  | Yes | No | NR |  |  |  |
| Ramis 2020 |  |  | √ | – | NR | 6.77% |
| Credeur 2019 |  |  | √ | – | 100% | 0 |
| Kambic 2019 |  | √ |  | – | 100% | 0 |
| Lopes 2019 |  | √ |  | – | 100% | 0 |
| Mouser 2019 |  |  | √ | – | NR | 5% |
| Barili 2018*** | √ |  |  | 2 (12.5%) | NR | 40% |
| Boeno 2018 |  |  | √ | – | NR | 0 |
| Karabulut 2018 |  |  | √ | – | NR | 0 |
| Natsume 2018 |  | √ |  | – | 100% | 0 |
| Sardeli 2017 |  |  | √ | – | 100% | 12.5% |
| Paiva 2016 |  | √ |  | – | 100% | 0 |
| Shimizu 2016 |  |  | √ | – | NR | 0 |
| Yasuda 2016 |  |  | √ | – | NR | 0 |
| Yasuda 2015a |  |  | √ | – | NR | 0 |
| Yasuda 2015b |  |  | √ | – | NR | 28% |
| Fahs 2014 |  | √ |  | – | NR | 0 |
| Ozaki 2013 |  |  | √ | – | NR | 12% |
| Fahs 2012 |  |  | √ | – | 100% | 0 |
| Hunt 2012 |  |  | √ | – | 100% | 0 |
| Larkin 2012 |  |  | √ | – | NR | 0 |
| Clark 2011 |  |  | √ | – | NR | 0 |
| Figueroa 2011 |  | √ |  | – | 100% | 0 |
| Patterson 2011 |  |  | √ | – | 100% | 0 |
| Credeur 2010 |  | √ |  | – | 100% | 0 |
| Patterson 2010 |  |  | √ | – | NR | 0 |
| Renzi 2010 |  |  | √ | – | NR | 0 |

–, Not applicable NR, Not reported; *, Percentage of exercise sessions attended by the subjects; **, Percentage of subjects who gave consent and were randomized, but did not have their data included in the analysis; ***, Reported adverse event was sensation of leg numbness in the first moments of exercise with BFR, which did not persist beyond the first minute of exercise.
