# Supplementary material for: Effects of exercise training with blood flow restriction on vascular function in adults: a systematic review and meta-analysis
Source: PeerJ. 2021 Jul 7;9:e11554. doi: 10.7717/peerj.11554 (PMC8272459; doi:10.7717/peerj.11554)
Supplement: Supplemental Information 8 [file peerj-09-11554-s008.docx]

1. The rationale for conducting the systematic review / meta-analysis;

Blood flow restricted exercise (BFRE) is a low intensity exercise modality and, while being commonly used within athletic training programs, has potential benefit and utility for populations that find it difficult to perform traditional exercise at an intensity high enough for maximal physiological adaptations. However, these populations (e.g., elderly, people with COPD) often present impaired vascular function raising the importance of understanding the effects of BFRE in vascular function.

While there is a number of systematic reviews regarding effects of BFRE on muscle strength and hypertrophy, aerobic capacity and blood pressure, very little is known regarding BFRE and vascular function, especially when it comes to the inclusion of comprehensive systematic review with meta-analysis and meta-regression models.

1. The contribution that it makes to knowledge in light of previously published related reports, including other meta-analyses and systematic reviews.

Studies that have investigated the effects of BFRE on VF have reported heterogenous results, with both beneficial and deleterious [21,22] effects reported for VF. Two systematic reviews were published regarding BFRE and VF, however they have identified only six [23] and five [24] studies, with one of them only including resistance training and focusing in just one aspect of VF (arterial stiffness) [24]. Thus, this study expands on previous manuscripts and performs a comprehensive systematic review with meta-analysis and meta-regression models aiming to explore the effects of BFRE on vascular function in adults with or without chronic health conditions.
