# Supplementary figures and images for: Effects of exercise training with blood flow restriction on vascular function in adults: a systematic review and meta-analysis
Source: PeerJ. 2021 Jul 7;9:e11554. doi: 10.7717/peerj.11554 (PMC8272459; doi:10.7717/peerj.11554)

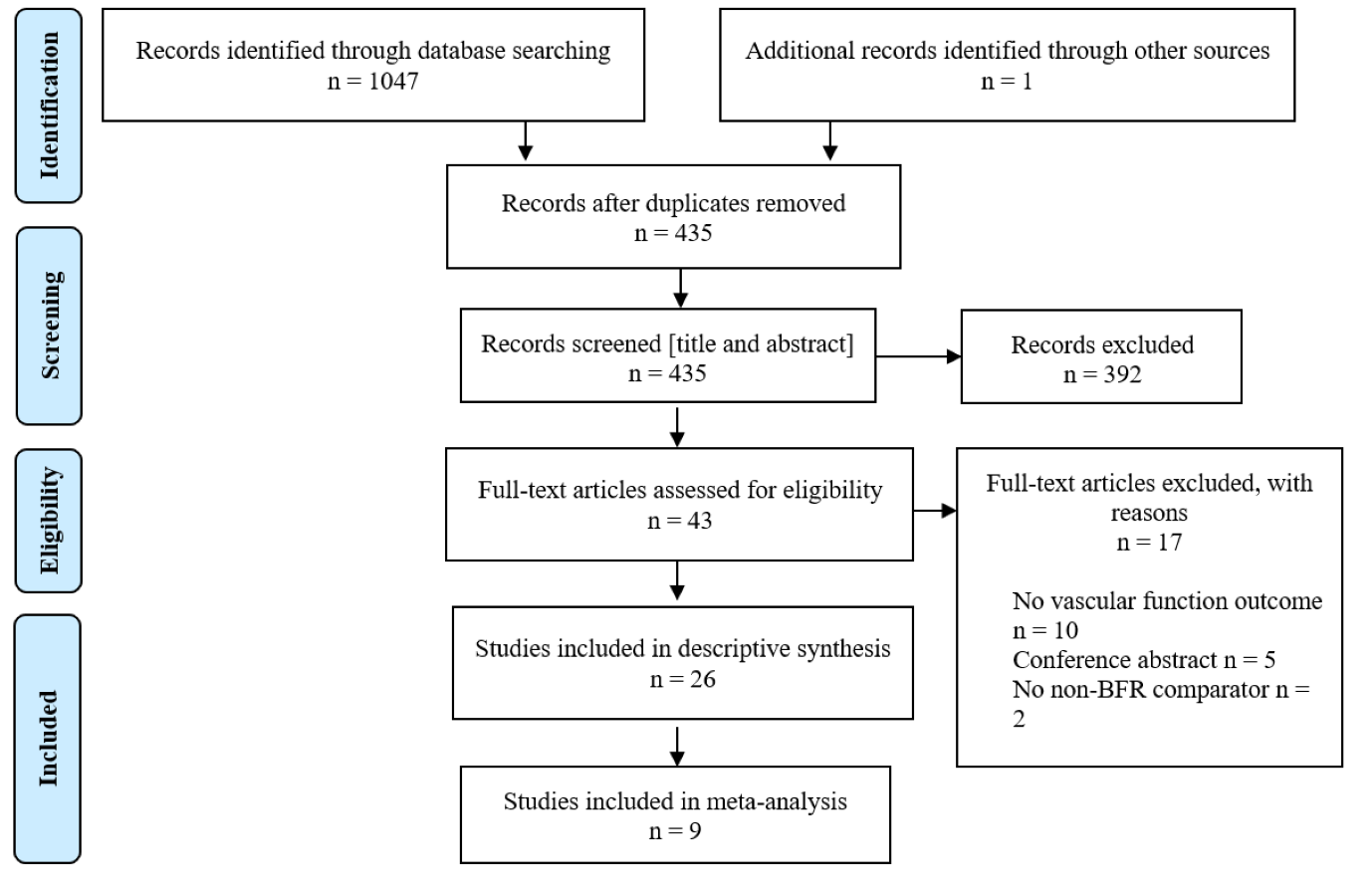

Supplement: Supplemental Information 10 [file peerj-09-11554-s010.png]
